# Your smartphone could act as a pulse-oximeter and as a single-lead ECG

**DOI:** 10.1038/s41598-023-45933-3

**Published:** 2023-11-06

**Authors:** Ahsan Mehmood, Asma Sarouji, M. Mahboob Ur Rahman, Tareq Y. Al-Naffouri

**Affiliations:** Department of Electrical Engineering, KAUST, Thuwal, Kingdom of Saudi Arabia

**Keywords:** Optical sensors, Biomarkers, Cardiology, Health care, Medical research, Computer science, Biomedical engineering

## Abstract

In the post-covid19 era, every new wave of the pandemic causes an increased concern/interest among the masses to learn more about their state of well-being. Therefore, it is the need of the hour to come up with ubiquitous, low-cost, non-invasive tools for rapid and continuous monitoring of body vitals that reflect the status of one’s overall health. In this backdrop, this work proposes a deep learning approach to turn a smartphone—the popular hand-held personal gadget—into a diagnostic tool to measure/monitor the three most important body vitals, i.e., pulse rate (PR), blood oxygen saturation level (aka SpO2), and respiratory rate (RR). Furthermore, we propose another method that could extract a single-lead electrocardiograph (ECG) of the subject. The proposed methods include the following core steps: subject records a small video of his/her fingertip by placing his/her finger on the rear camera of the smartphone, and the recorded video is pre-processed to extract the filtered and/or detrended video-photoplethysmography (vPPG) signal, which is then fed to custom-built convolutional neural networks (CNN), which eventually spit-out the vitals (PR, SpO2, and RR) as well as a single-lead ECG of the subject. To be precise, the contribution of this paper is twofold: (1) estimation of the three body vitals (PR, SpO2, RR) from the vPPG data using custom-built CNNs, vision transformer, and most importantly by CLIP model (a popular image-caption-generator model); (2) a novel discrete cosine transform+feedforward neural network-based method that translates the recorded video-PPG signal to a single-lead ECG signal. The significance of this work is twofold: (i) it allows rapid self-testing of body vitals (e.g., self-monitoring for covid19 symptoms), (ii) it enables rapid self-acquisition of a single-lead ECG, and thus allows early detection of atrial fibrillation (abormal heart beat or arrhythmia), which in turn could enable early intervention in response to a range of cardiovascular diseases, and could help save many precious lives. Our work could help reduce the burden on healthcare facilities and could lead to reduction in health insurance costs.

## Introduction

One flip-side of the covid19 pandemic is that it has sparked great interest among people to stay informed about their overall well-being. Such interest in self-examination of one’s own body vitals hikes whenever a new wave of covid19 strikes the world. By now, it is well known that the important body vitals deviate from their nominal values when one is infected with covid19^1,2^ For example, the body temperature of a covid19 patient is often elevated ($100^{\circ }\,{\text {F}}$ or more). Additionally, a blood oxygen saturation level (aka SpO2) of less than 90% and a respiratory rate of less than 15 and more than 25 typically also indicate a possible covid19 infection. Last, but not the least, an elevated pulse rate may also be witnessed as an occasional side-effect of covid19. Need not to say, but the body vitals have huge clinical significance other than covid19 detection as well. For example, the deviation of RR from nominal value could indicate the following: cardiac arrest^3^, respiratory dysfunction^4^, pneumonia^5^, lungs cancer^6^.

Thus, enabling masses to self-measure their body vitals in a rapid and non-invasive manner using low-cost, portable equipment (e.g., smartphones, smart watches, wristbands, etc.) is an objective of key importance, as suggested by the World Health Organization (WHO)^7^. To this end, note that there are currently more than 6 Billion active smartphone users, and this number will rise to 7 Billion by 2025^8^. Therefore, it is only logical to devise mechanisms that exploit the onboard sensors and computational capability of modern-day smartphones to help realize the patient-centric healthcare systems of tomorrow.

In fact, there has been a recent surge of interest in smartphone-based estimation of body vitals, by extraction of various kinds of physiological signals. To this end, approaches that could extract photoplethysmography (PPG), electrocardiography (ECG), and phonocardiography (PCG) signals from the smartphone have been explored the most. PPG methods utilize the camera of the smartphone to measure the quantity of visible light reflected off the fingertip. A review of the PPG techniques and their potential applications could be found in Refs.^9,10^. The ECG methods compute the electric potential difference across a pair of external electrodes mounted on the casing of the smartphone^11^ (when one places his/her thumbs on the two electrodes). Finally, the PCG methods try to utilize the microphone of the smartphone to listen to faint sounds generated by the heart during each cardiac cycle.

**Figure 1:**
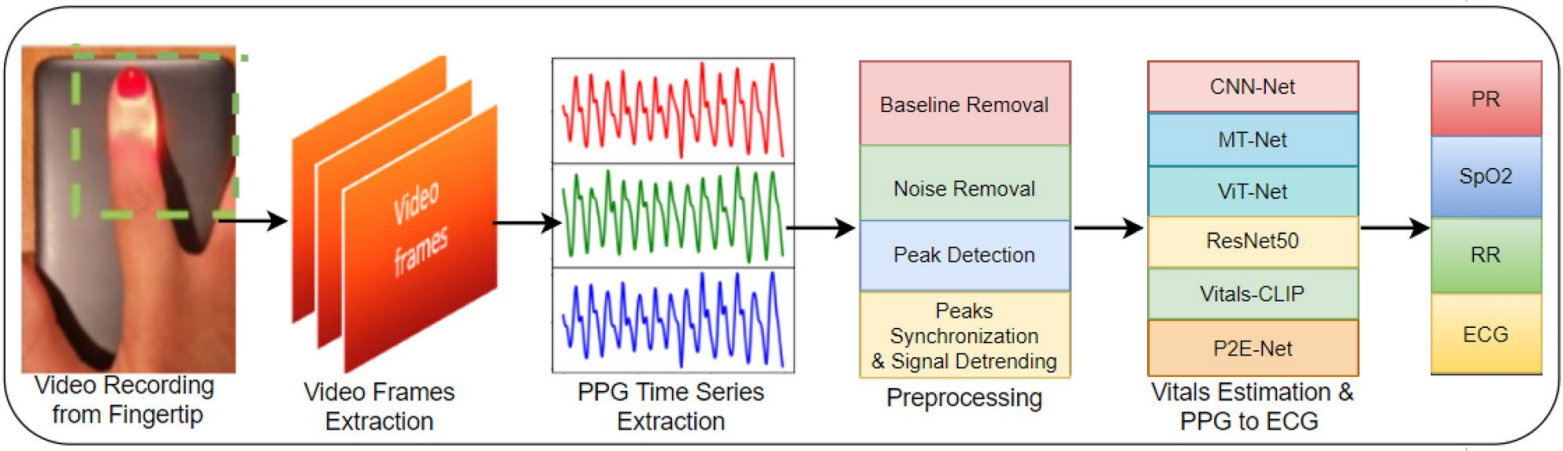
A quick graphical summary of this work.

In this pretext, we propose the utilization of deep learning techniques on PPG data derived from a smartphone to create a physiological monitor that can aid in the self-assessment of body vitals. Our study aims to evaluate the accuracy, robustness, and generalization capability of several deep learning models, including convolutional neural networks (CNNs), vision transformer (ViT), and CLIP model (that generates captions of given images), for the estimation of vitals and the reconstruction of electrocardiograms (ECGs). The core steps of our work are depicted in Fig. 1 and are elaborated on as follows:

- *Video recording* one needs to place his/her index finger on the rear camera of the smartphone in order to record a small video snippet of a small duration (say, 30 s).
- *Video preprocessing* On RGB (red, green, blue) channels of video, pixel-averaging on each frame is done to get a PPG time series, and Wavelet transform is applied for denoising and removal of motion-induced artifacts.
- *Training and testing of custom-built neural networks* once the PPG time series is available, it is passed to a number of deep neural networks which eventually spit out the vitals (PR, SpO2, and RR) as well as a single-lead ECG of the subject.

### Outline

The rest of this paper is organized as follows. “Related work” section describes selected related work. “Datasets” section discusses PPG and video PPG datasets including our two custom datasets. “Vitals estimation using video-PPG” section describes the essential details of various neural network models for vitals estimation (including a vision transformer as well as CLIP caption selector model) that are trained on several datasets. “Single-lead ECG synthesis from video-PPG” section discusses the architecture of the proposed P2E-Net, which translates a video-PPG signal acquired from a smartphone into a single-lead ECG signal. “Discussion” section concludes the paper.

## Related work

The objective of this study is to extract a PPG signal from video-PPG data, which is obtained by recording a video while placing the index finger on the rear or front camera of a smartphone. To achieve this, we conduct a comprehensive review of existing research that estimates three body vitals—pulse rate (PR), oxygen saturation (SpO2), and respiratory rate (RR)—using both video-PPG signals and traditional PPG signals acquired via a pulse oximeter. Additionally, we provide a brief overview of the latest techniques for acquiring single-lead ECG using a smartphone.

### Video PPG-based vitals estimation

Several studies have explored the use of video-PPG data to estimate body vitals, and a summary of these works is presented in Table 1. However, the table highlights the limited availability of publicly accessible datasets for this purpose. The existing datasets either have a small number of examples or only provide labeled data for a subset of the vitals of interest. In contrast, this study aims to estimate not only the three vitals (pulse rate, oxygen saturation, and respiratory rate) but also the single-lead electrocardiogram (ECG), which is not addressed in the existing works. (Note that there are works under the name remote PPG or iPPG that measure the body vitals using dedicated cameras to record the face video and extract PPG signal from the video (this line of work was popular before the advent of smartphones), see^12^ and references therein. Moreover, there is a recent flux of smart watches/smart wristbands (by Apple, Samsung, Fitbit, etc.) capable of measuring the body vitals. But since this work investigates the feasibility of smartphones as a physiological monitor, discussion of these works is out of the scope of this work.)

**Table 1:** Quick comparison of our work with the most relevant related works (all works utilize video-PPG data from the fingertip).

Ref.	Vitals measured	Dataset	Methodology
^13^	PR, SpO2, BP	Private	Peaks detection
^14^	SpO2	Private	SVD + CNN
^15^	SpO2	Private	−
^16^	PR	BUT PPG	−
^17^	PR, PRV	Welltory	Wavelet analysis
^18^	HR, SpO2	MTHS	CNN
^19^	HR, HRV, RR, SpO2	–	Peaks detection, VFCDM
This work	HR, SpO2, RR, ECG (single-lead)	BIDMC, MTHS, PulseDB and custom	CNN, DCT+FFNN

*PRV (HRV)* pulse (heart) rate variability, *BP* blood pressure, *HR* heart rate.

#### Pulse rate

The estimation of pulse rate has traditionally relied on specialized sensors that record PPG signals. However, later studies have explored the potential of smartphones to record vPPG (alternatively called video-PPG) signals and estimate vital signs. Early research, such as^19^, proposed using smartphones to estimate HR (alternatively called PR), and since then, smartphones have become widely studied as devices for measuring vital signs. Researchers have utilized various sensors available on smartphones to monitor vitals. For instance, Ref.^20^ proposed using a gyroscope to measure HR, while^13,21,22^ used the rear camera of the mobile phone to measure pulse rate using fingertip videos. The front camera has also been used to measure HR, as in Ref.^23^. The vPPG signals have been recorded from various body locations, such as the face^24,25^, fingertips^13,21,22^, and forehead^26^ to extract vPPG for pulse rate estimation. Moreover, numerous methods have been proposed to measure vitals from vPPG signals, such as using Fourier transform and peak detection-based algorithms for pulse rate estimation in Ref.^27^ and principal component analysis (PCA) in Ref.^28^. Some researchers have also employed deep learning (DL) models for pulse rate estimation, such as using a CNN in Ref.^29^ and long short-term memory (LSTM) based attention network in Ref.^30^. Recently, researchers have explored the use of various transformer-based models for pulse rate estimation, such as vision transformer^31^, TransPPG^32^, and Radiant^33^.

#### Blood oxygen saturation (SpO2)

Since the smartphone cameras are not designed for pulse oximetry, smartphone-based SpO2 estimation faces several challenges, e.g., lack of infrared LED, lack of a well-accepted mathematical model, noisy vPPG signal, variable placement and pressure of fingertip on camera lens^34^. Thus, authors in Ref.^34^ provide add-on hardware to measure SpO2 whose results meet the food and drug authority (FDA) accuracy standard when tested on six subjects only. Another smartphone-based pulse oximeter solution based on meta region of convergence (MROI), the ratio of ratio (RoR), and linear regression method are presented in Ref.^35^. This method though that does not require any additional hardware has the limitation that it cannot estimate low SpO2 values. One of the earliest works utilizing the RoR method for mobile-based SpO2 estimation is^19^; however, its accuracy is not up to the mark based on the FDA clearance threshold as it is also not able to estimate low SpO2 levels. For the patients of respiratory disease, a SpO2 estimation algorithm for low SpO2 level detection is presented in Ref.^15^. In Ref.^36^ RoR and linear regression method is used to measure SpO2. The RoR method uses green and red channel wavelength, the amplitude of the vPPG from different channels are different and it depends on the quantum efficiency of the camera for different wavelengths. Thus, Ref.^37^ incorporated camera quantum efficiency in the measurement of SpO2 using a smartphone. Although the performance of this method is improved it suffers from a limitation of the unavailability of quantum efficiency of mobile cameras. To mitigate this problem, recently, authors in Ref.^13^ proposed a method that neither requires information of quantum efficiency nor dedicated external hardware for the measurement of SpO2. Last but not least, the authors in Ref.^14^ estimated SpO2 using convolutional neural networks.

#### Respiratory rate

**Figure 2:**
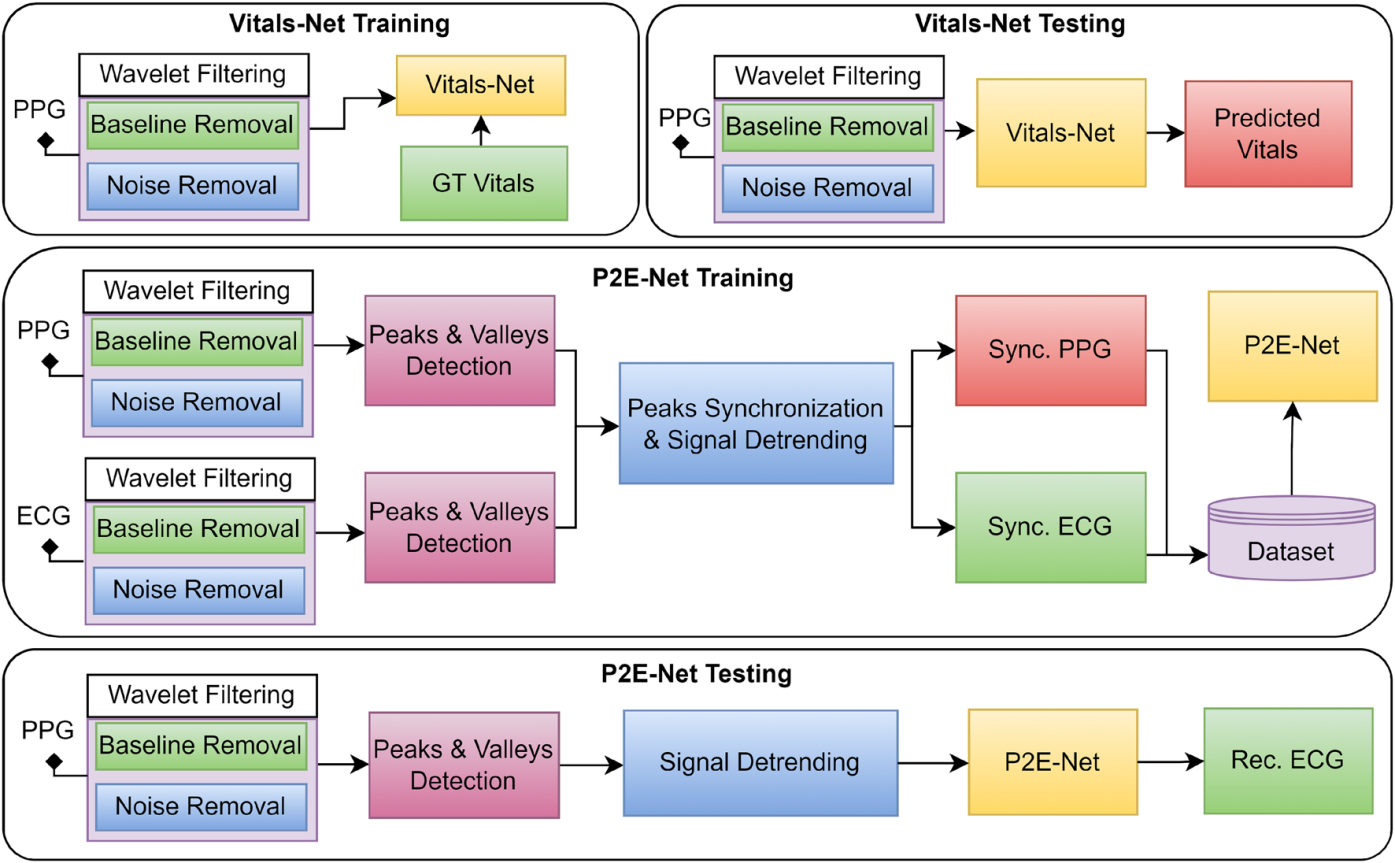
Highlights of the pre-processing done during the training and testing phases of: (1) vitals estimation, and (2) vPPG-to-ECG translation.

Respiratory rate measurement has traditionally been carried out by manually counting chest movements, a time-consuming and inaccurate process^38^. Additionally, medical-grade equipment for RR measurement is costly and not widely available for use in wearable mobile devices^39^. However, mobile phones have become increasingly popular for measuring RR using various sensors. For instance, Ref.^40^ utilized the built-in microphone of a mobile phone to record nasal breath sounds for RR estimation. Similarly, Ref.^41^ measured RR using video recorded from the fingertip using a mobile phone’s rear camera, utilizing three different methods: autoregressive model (AR), variable-frequency complex demodulation (VFCDM), and continuous wavelet transform (CWT). Aly et al.^42^ utilized the accelerometer and gyroscope of a mobile phone held on a human chest to extract RR, while Ref.^43^ used the discrete wavelet transform to measure RR from video recorded from the fingertip, extracting the vPPG. Moreover, several studies have investigated deep learning (DL)-based approaches for RR estimation, with Shuzan et al.^44^ recently investigating 19 DL models for estimating RR and HR, with the Gaussian process regression model demonstrating the best performance (Fig. 2).

### Single-lead ECG reconstruction

The clinical significance of a single-lead ECG is well-known as it allows atrial fibrillation (i.e., abnormal/irregular heartbeat) detection. Nevertheless, there exist only a couple of works that have considered the generation of a single-lead ECG signal using a smartphone and/or a pulse-oximeter. For example, Ref.^11^ mounts two dry electrodes on the back-casing of the smartphone in order to measure the single-lead ECG by placing one’s both thumbs on the two dry electrodes. Reference^45^ passes the PPG signal acquired from a pulse oximeter through a block that computes the discrete cosine transform, followed by ridge regression, in order to get the single-lead ECG signal. Reference^46^ also attempts to translate a PPG signal (acquired by a pulse oximeter) to a single-lead ECG signal, but by using a conditional generative adversarial network (c-GAN).

### Research gap

To the best of the authors’ knowledge, a stand-alone smartphone-based solution that measures the body vitals (PR, SpO2, RR), as well as a single-lead ECG (without any external add-on hardware/sensors), has not been discussed in the open literature to date. Moreover, existing video-PPG-based frameworks are optimized for some specific scenarios on small datasets. Work on the generalization capability and robustness of the DL-based vitals estimation model is scarce.

### Contributions

Having motivated the research gap, the main contribution of this paper is twofold:

- *Estimation of body vitals* We pre-process the video-PPG signal acquired from a smartphone and feed it to various custom-built DL models (including a vision transformer and a CLIP model) which eventually output the three most important body vitals (PR, SpO2, RR). We do hope that our dataset will serve as a benchmark dataset to test the generalization capability, accuracy and robustness of future deep learning methods for vitals estimation.
- *Synthesis of single-lead ECG* We pre-process the video-PPG signal acquired from a smartphone and utilize a novel discrete cosine transform+feedforward neural network-based method that translates the recorded video-PPG signal to a single-lead ECG signal. To the best of our knowledge, this is the first work that reconstructs a single-lead ECG from a video-PPG signal acquired from a smartphone.

*Significance of this work* The significance of our work is twofold:

- This work enables rapid self-testing of various important body vitals (i.e., heart rate, respiratory rate, blood oxygen saturation level) using one’s own smartphone. This opens the door for the realm of patient-centric healthcare whereby the chronic patients, palliative care patients, people in remote areas (mountains, deserts), people in marine/sea expeditions, people in harsh climates (e.g., arctic) could monitor and keep track of their body vitals. The proposed method could also revolutionalize the symptom monitoring methods for covid19 suspects and patients.
- This work enables an off-the-shelf smartphone to provide a single-lead ECG. Thus, it enables rapid self-testing for atrial fibrillation detection using one’s own smartphone. Once atrial fibrillation is detected by the smartphone, it will prompt the user to visit a hospital. Since atrial fibrillation is a risk factor for stroke and heart failure, a timely detection of atrial fibrillation could in turn lead to early diagnosis of a range of cardiovascular diseases at a tertiary healthcare facility. Therefore, the proposed method has the potential to reduce the burden at healthcare facilities. Additionally, early diagnosis of a variety of cardiovascular diseases will lead to early intervention by the cardiac physicians which will in turn to lead to reduction in health care (and health insurance) costs, and will potentially save many precious lives.

## Datasets

In this subsection, we outline the details of our K20-vPPG and K1-vP2E datasets. But before that, it is imperative to provide the reader with a systematic and brief review of the relevant existing public datasets on PPG and vPPG (as well as their limitations). (Note that there are some PPG datasets that are constructed using commercial pulse oximeters, e.g., MIMIC-III dataset^47,48^, video-PPG datasets based upon traditional cameras^12^ (before the advent of smartphones), and video-PPG datasets that utilize PPG signals for biometrics/authentication purposes, e.g., biosec1 dataset^49^. But since this work primarily focuses on video-PPG datasets acquired using smartphones for vitals estimation, discussion of such datasets (and the corresponding works) are out of the scope of this work.).

### Existing video-PPG datasets

#### BUT-PPG dataset^16^

This dataset contains 48 simultaneous records of video-PPG data and single-lead ECG data of 12 healthy subjects (6 males, 6 females), 21 to 61 years old. Each video of the index finger of the subject was recorded for a duration of 10 s, using a Xiaomi MI9 smartphone at a frame rate of 30 Hz. The single-lead ECG data were recorded using a Bittium Faros 360 device at a sampling rate of 1000 Hz and were manually annotated by an expert. Eventually, Ref.^16^ utilized their dataset to estimate the HR.

#### Welltory dataset^17^

This dataset contains 21 records of video-PPG data of 13 healthy subjects, 25 to 35 years old. Each video of the index finger of the subject was recorded for a duration between 1 and 2 min, using the Welltory android app. The R-peak to R-peak (RR) intervals were recorded using a Polar H10 ECG chest strap and were manually examined by an expert. Eventually, Ref.^17^ utilized their dataset to estimate the HR and HRV.

#### MTHS dataset^18^

This dataset contains 65 recordings of video-PPG data along with corresponding HR and SpO2 labels, of 62 patients (35 males, 27 females). Each video of the index finger of the subject was recorded using an iPhone 5s smartphone at a frame rate of 30 Hz. The ground truth/labels were obtained using a pulse oximeter (M70) at a sampling rate of 1 Hz. Eventually, Ref.^18^ utilized their dataset to estimate the HR and SpO2.

#### Limitations of existing vPPG datasets

We identified some limitations/shortcomings of the aforementioned vPPG datasets, some of them are as follows. The BUT-PPG dataset provides labels for HR only, while the Welltory dataset provides labels for HR and HRV only. Moreover, the small size of BUT-PPG and Welltory datasets renders them infeasible for state-of-the-art but data-hungry deep learning methods. The MTHS dataset though contains sufficient examples (enough to train a neural network) but provides labels for HR and SpO2 only. More importantly, the need for a new and large dataset on video-PPG stems from the fact that the generalization capability, accuracy, and robustness of any deep learning algorithm for vitals estimation could only be tested if a handful of video-PPG datasets are publicly available.

### Benchmark PPG datasets

#### The BIDMC dataset

As we mentioned earlier in this section, one could learn more about the generalization capability, accuracy, and robustness of his/her proposed deep learning algorithm by testing it on other datasets (with unseen data with potentially different distributions). Therefore, this work utilizes the well-known BIDMC dataset^50^ (in addition to the K20-vPPG dataset) for the training and performance evaluation of the proposed method for vitals estimation. Some most pertinent details of the BIDMC dataset are as follows. The BIDMC dataset contains 53 sessions (each of duration eight minutes) of simultaneously recorded PPG and ECG signals, along with the ground truth values (i.e., the vitals). The PPG and ECG signals are recorded at a sampling frequency of 125 Hz, whereas the ground truth values of HR, SpO2, and RR are recorded at a sampling rate of 1 Hz. Note that the PPG signals in this dataset were acquired from the fingertip of patients using the clinical pulse oximeter. Finally, the single-lead ECG signal collected in this dataset is the Lead-II acquired using the standard 12-lead ECG.

#### The PulseDB dataset^51^

This dataset contains a large number of filtered PPG and ECG signals. It also contains the ground truth labels for HR and BP. We randomly download the data of 550 subjects both male and female with more than 16,000 PPG, and ECG signals along with their corresponding vitals ground truth labels. Each PPG and ECG signal is 10 s long and sampled at 125 Hz. The corresponding ground truth labels are recorded at 1 Hz.

### Our video-PPG datasets

#### K20-vPPG dataset

The limitations of the existing vPPG datasets (e.g., a small number of training examples, lack of raw data and labels for RR, and single-lead ECG, as needed by our study) prompted us to run an extensive campaign for vPPG data collection of our own. Thus, we subsequently compiled a new dataset named K20-vPPG dataset. The data collection campaign was approved by the ethical institutional review board (EIRB) of our institution, and all the subjects voluntarily participated in this data collection activity. Next, we discuss all the relevant details of the K20-vPPG dataset.

##### Participants

A total of 20 healthy subjects with no history of cardiac or respiratory disease participated in this data collection campaign, of which 5 were females and 15 were males. The volunteers/participants were either employees or students at our institute, aged 16–36 years.

##### Data characteristics

For each subject, we recorded the 2 to 10 min long vPPG data (the raw data) from the index fingers of the right hands of twenty different subjects. For ground truth/labels for supervised learning later, we simultaneously recorded the three body vitals (PR, SpO2, RR). (Our dataset also contains the ground truth labels of perfusion index (Pi) and Pleth Variability Index (PVi). However, their discussion is out of the scope of this paper.)

#### K1-vP2E dataset

**Figure 3:**
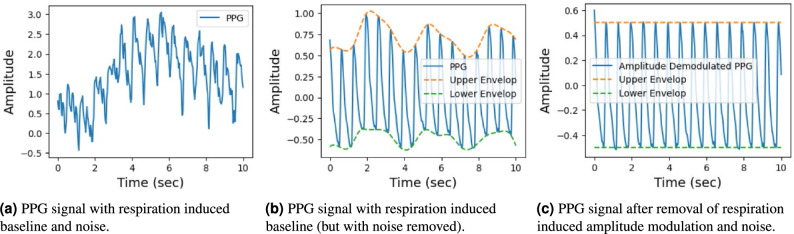
Detrending and denoising: two key steps in the preprocessing of a PPG signal.

For training the P2E-Net, the lead author simultaneously recorded 24, 5–10 min long vPPG and 1-lead ECG signals of himself over a time period of seven days after different activities (e.g. eating, running, sleeping, and walking). Then, the raw PPG and ECG signals were filtered and detrended in a similar way, as shown in Fig. 3.

## Vitals estimation using video-PPG

The main objective of this section is to conduct a comprehensive evaluation of DL-based models for vitals estimation, focusing on their generalization capability, accuracy, and robustness. Previous studies have highlighted that existing video-PPG and DL-based approaches are extensively parameterized and optimized on small public datasets. While these models may exhibit strong performance on these datasets, they often lack the ability to generalize and demonstrate robustness in diverse scenarios. Thus, our contribution lies in enhancing the generalization capability and robustness of various DL-based architectures. The section begins by discussing crucial pre-processing steps, followed by a detailed description of our proposed DL-based model, Vitals-Net. Finally, we conclude this section with a thorough performance evaluation of our DL models.

### Pre-processing stage

The pre-processing of the PPG signal extracted from the video-PPG data is performed prior to training the vitals-Net model. Careful pre-processing not only enhances the training accuracy of a neural network but also facilitates the model’s training process. Our pre-processing stage has been specifically designed to mitigate various distortions, e.g., baseline drift, high-frequency noise, artifacts caused by ambient light and motion, and the de-trending of the PPG signal. The following are the detailed pre-processing steps (also summarized in Fig. 2).

#### PPG signal extraction from video-PPG

The pixel averaging technique is employed to extract a PPG time series from the central region of each frame in the video-PPG. This yields a vPPG signal, which is combined with simultaneously recorded ground truth vital signs obtained from an oximeter for training and validation purposes.

#### Sliding window mechanism for segmentation

We then do segmentation of the PPG data. Since the ground truth labels for vitals are recorded at a frequency of 1 Hz, a sliding window mechanism is utilized. This mechanism uses a stride of 1 s, and allows us to creation a large labelled PPG dataset with 4330 examples or segments.

#### Wavelet filtering

Next, the vPPG signal undergoes wavelet filtering to eliminate low-frequency baseline drift, high-frequency noise, artifacts induced by ambient light, and motion artifacts. A five-level decomposition based on fast wavelet transform is applied to the PPG signal to obtain approximate and detailed coefficients. Subsequently, the signal is reconstructed by selectively choosing appropriate wavelet coefficients, as described in Ref.^52^. The low-frequency baseline induced by respiration and changes in ambient light intensity is represented by the signal corresponding to the approximate coefficients. The high-frequency noise and motion artifacts are represented by the signals corresponding to the level 1 and 2 detailed coefficients, respectively. Therefore, the reconstructed signal consists of wavelet coefficients other than the approximate and detailed coefficients from the first two levels of decomposition. Figure 3a illustrates the input raw PPG signal, while Fig. 3b shows the corresponding filtered signal. Finally, Fig. 3c showcases the de-trended and de-noised PPG signal, which is particularly useful for ECG reconstruction.

### Vitals-Net models and training

#### Vitals-Net models

We train four different models namely CNN-Net, MT-Net, ViT-Net, and ResNet, to estimate the vitals. Additionally, we train the Vitals-CLIP model for querying-based vitals estimation.

##### CNN-Net model

This model is a variant of a CNN model proposed in Ref.^53^ which is originally proposed for heart rate estimation; however, we fine-tuned this model to estimate all vitals. Specifically, we add a lambda layer, that computes a short-time Fourier transform of the input PPG signal, at the top of this model. Moreover, we add batch normalization followed by a dropout layer after the flattening layer and each dense layer to avoid over-fitting and ease the training process. We trained four different models: CNN-Net, MT-Net, ViT-Net, and ResNet, for vital sign estimation. Additionally, we trained a Vitals-CLIP model for querying mode vitals estimation.

##### MT-Net model

This model is another fine-tuned model originally proposed for HR and SpO2 estimation using vPPG^54^. To get the best performance we add a lambda layer, that computes the DCT of input, on top of the model followed by an ADD layer, which adds the input in the output of the DCT lambda layer. Moreover, we remove the last conv1D and replace it with an FC layer with “relu” activation.

##### ViT-Net model

**Figure 4:**
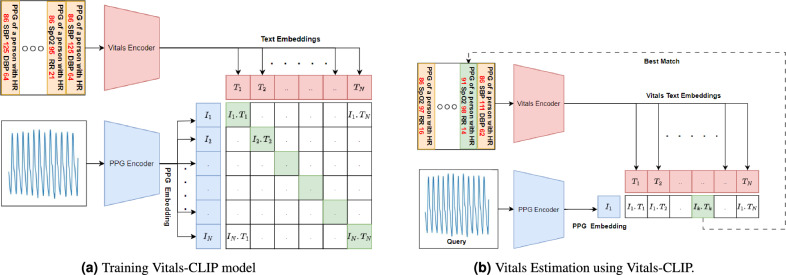
The CLIP neural network model for one-shot estimation of Vitals.

This model is a fine-tuned vision transformer. For compatibility, we added a lambda layer capable of performing a short-time Fourier transform of the input PPG signal. It is worth noting that the ViT model inherently expects a 2D vector/matrix as input, and the short-time Fourier transform converts the PPG signal into a suitable 2D vector/matrix format, making the lambda layer appropriate for integration with the ViT model (Fig. 4).

##### Vitals-CLIP model architecture

**Figure 5:**
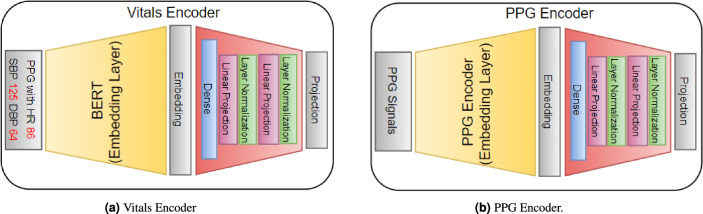
The model architecture details of encoders in Vitals-CLIP. The inner architecture of the PPG encoder’s embedding layer and linear projection layer is given in Table 2.

Vitals-CLIP is an enhanced version of the audioCLIP model^55^, which extends its capabilities by incorporating PPG signals along with the text. To achieve this enhancement, we integrate specialized encoder models for PPG and text encoding, which consists of a text embedding layer followed by a projection layer (see Fig. 5a) and PPG embedding layer followed by a projection layer (see Fig. 5b), into the existing CLIP framework (see Fig. 4), leveraging the vPPG and PulseDB dataset. This integration allows our proposed model to perform both bimodal and unimodal querying tasks while maintaining CLIP’s impressive generalization capability to novel datasets.

**Table 2:** Model architecture of PPG Encoders in CLIP.

Layer	PPG embedding			Linear projection	
	Type	Output	($N_f$, $K_s$,*s*)	Layer	Output
1	Input	(1250, 1)	–	GELU	(256)
2	Conv1D	(1241, 8)	(64, 10, 1)	Dense	(256)
3	MaxPooling1D	(620, 8)	–	Dropout	(256)
4	Conv1D	(616, 8)	(32, 5, 1)	ADD	(256)
5	MaxPooling1D	(308, 8)	–		
6	Conv1D	(306, 16)	(16, 3, 1)		
7	Conv1D	(304, 32)	(8, 3, 1)		
8	Flatten	(9728)	–		

Here, $N_f$, $K_s$, and *s* denote the number of filters, kernel size, and stride respectively.

The model comprises three encoder models: Vitals Encoder, PPG Encoder, and ECG Encoder. The Vitals Encoder consists of a text encoder, that generates embeddings of the text input, followed by an embedding projection model. We utilize a pre-trained BERT model available at TensorFlow Hub, that could be downloaded from the following URL: https://tfhub.dev/tensorflow/small_bert/bert_en_uncased_L-4_H-512_A-8/1. The embedding projection layer in the Vitals Encoder includes a Dense layer, followed by two linear projection layers. Each linear projection layer consists of a ‘gelu’ activation layer, a dense, a dropout layer with a dropout rate of 0.2, an ADD layer that adds the output of the dropout layer and the first dense layer outside the linear projection layer, and a ’layernormalization’ layer at the end.

The PPG encoder consists of a 1D CNN-based model, that outputs low dimensional embeddings of both PPG and ECG signal, followed by the same embedding projection layer described above. More specifically the architecture of the PPG encoder is summarized in Table 2.

#### Training

We train our models in three different configurations *Vital Specific Training Configuration—*From the literature survey, it is clear that training and testing of DL models are performed independently for each vital. Therefore, we train our models first in a vital specific configuration. In this configuration, we train our models for each individual vitals. In this configuration, we train our model using a video PPG dataset and then by BIDMC dataset. The video PPG dataset contains 21–30, 5–10 min long vPPG signals that were obtained by averaging each frame of fingertip video. Similarly, the BIDMC contains 8 min long each from 53 subjects. To train these models we segment each signal in BIDMC and K20-vPPG datasets in a vital specific window size. For example, we segment each PPG using $w_s = 10$ s for vitals HR and RR and $w_s = 30$ s for RR. For each segment, we use an average of 10 labels for HR/SpO2 and an average of 30 RR labels for RR estimation. By the segmentation, we get a dataset $D \in {\mathscr {R}}^{N_v, N \times w_s*Fs \times ch }$ where *N*, $N_v$ and *ch* denotes the number of PPG and label pairs, number of vitals and the number of channels, e.g. $ch = 2$ means red and green video PPG signals, respectively. The dimensions of BIDMC and vPPG datasets are ${\mathscr {D}}_b = {\mathscr {R}}^{\{N_v, 22,550 \times 30 \times w_s \times 1\}}$ and ${\mathscr {D}}_v = {\mathscr {R}}^{\{N_v, 8890 \times 30 \times w_s\times 2\}}$ respectively. Other training parameters are added in Table 5. It is well known that PPG datasets are prone to outliers due to the estimation error of oximeters, therefore these models must be trained in such a way that they train robustly on datasets that contains some outliers. Fortunately, mean absolute errors (MAE) are known to be robust to outliers. Therefore, we train our models using the MAE loss function defined below,

1 $$\begin{aligned} {\mathscr {L}} = \frac{1}{|{\mathscr {B}}| }\sum _{{\textbf{y}} \in {\mathscr {B}}} |{\textbf{y}}-{\hat{{\textbf{y}}}}|, \end{aligned}$$

where ${\mathscr {B}}$ is the batch size, ${\textbf{y}}$ is the label, ${\hat{{\textbf{y}}}}$ is the prediction. With the above loss function, we train the model for a maximum of 1000 epochs, however, we apply an early stopping to avoid overfitting of models.

##### Joint training configuration

In many scenarios where these vitals estimation is performed using low resources devices e.g., wristbands, smart watches, or even mobile, estimation of each vital with different DL models is not resource efficient. Therefore, in contrast to the first configuration where we trained models for only one vital, in this configuration we stack all vitals in a vector and use that as a label while training the model. Similar to the previous configuration, in this configuration DL models are trained on both BIDMC and vPPG datasets independently. In contrast to the previous configuration, the $w_s$ is the same for all vitals. In this configuration, we use $w_s = 20$ s. The number of PPG and labels paired in the BIDMC and vPPG datasets are the same as the previous configuration.

##### CLIP configuration

In contrast to the previous two configurations, this configuration is specifically related to the Vitals-CLIP model and differs from the previous configurations in a number of ways. Firstly, this configuration involves a so-called pretraining which is totally different from the training first two configurations. Secondly, this configuration uses text captions (see Figs. 4a, 7) as labels rather than numeric labels. Thirdly, the pretraining is not dataset specific rather three datasets namely vPPG, BIDMC, and PulseDB, are used simultaneously in the pretraining. This is possible due to the fact that text captions can be of different sizes. We train Vitals-CLIP in this configuration on a very large dataset. To make the dataset larger we made use of the subset of the PulseDB dataset in addition to BIDMC and video PPG. We used a large number of 10 seconds long PPG signals from 550 subjects taken randomly from the PulseDB dataset and concatenate it with the BIDMC dataset containing the PPG of 53 subjects and also concatenated the video PPG dataset with them. Using the ground truth values of vitals, we made captions corresponding to each PPG segment. Finally, we get a dataset ${\mathscr {D}}_C \in {\mathscr {R}}^{\{N\times N_c,N \times w_s*F_s \times 1 \}}$ where $N = 38,600$, $N_c$ is caption length, $F_s = 125$ Hz and $w_s = 20$ s.

We use this dataset for the training of Vitals-CLIP. After the pretraining of Vitals-CLIP, we use it in querying mode (see Fig. 4b) due to its inherent ability to operate in querying mode. After pretraining we generate text embedding for all labels in the validation dataset and search for a caption using a vPPG signal as a query. The caption searching is performed by generating an embedding using a pre-trained PPG encoder and then taking the dot product with all caption/text embeddings. Then, the corresponding caption of the top *k* dot products are selected as captions (see Fig. 7). Then from all *k* captions, the values of vitals are extracted using a Python function, an average value is computed across the *k* captions, and predicted average value is compared against the ground truth labels. Finally, an important note here. After pre-training of Vitals-CLIP model, we generate a text embedding using a pre-trained text-encoder. These text embeddings are generated for the whole validation dataset that contain labels corresponding to three different datasets: (i) PulseDB, (ii) BIDMC, and (iii) k20-vPPG. Since PulseDB dataset contains labels for blood pressure as well, occasionally one or more of the top 3 captions generated by the Vitals-CLIP model might contain predicted labels of systolic blood pressure (SBP) and diastolic blood pressure (DBP), in response to the vPPG query signals from our K20-vPPG dataset. These labels are simply ignored, since K20-vPPG dataset does not contain ground truth labels for the SBP and DBP. Then, from the rest of the captions, predicted values of HR, SpO2 and RR are extracted, averaged across the three captions, and compared with the ground truth labels.

### Performance evaluation

To investigate the generalization capability of our DL models, we train them with K20-vPPG and BIDMC datasets and then test them on the LESSO data of both datasets. It is well-known that the performance evaluation using LESSO data of video-PPG is a superior measure of the generalization capability of a DL model^53^. Therefore, we left some subjects out (LESSO) to later use them for the testing of our trained DL models. Specifically, we left data of 5 subjects (randomly selected from each dataset) that then serves as LESSO data.

We begin with the K20-vPPG dataset and do exhaustive search to figure out the best vPPG signal length denoted by $w_s$ (window size) that minimizes the MAE for all vitals. Figure 9 shows that the MAE for parameters HR and SpO2 increase slightly with the increase in widow size because the ground truth labels are the average of all labels in that window size. Overall, the window size of 4 s proves to be the best window size for HR and SpO2 Estimation. The standard deviation in absolute error (SAE) fluctuates slightly but remains under 1.5. In contrast, we use a higher window size due to the fact that RR induces slow variations in the video-PPG signal. Both the MAE and SAE increase slightly and then decrease reaching their lowest values at a window size of 32 s.

**Figure 6:**
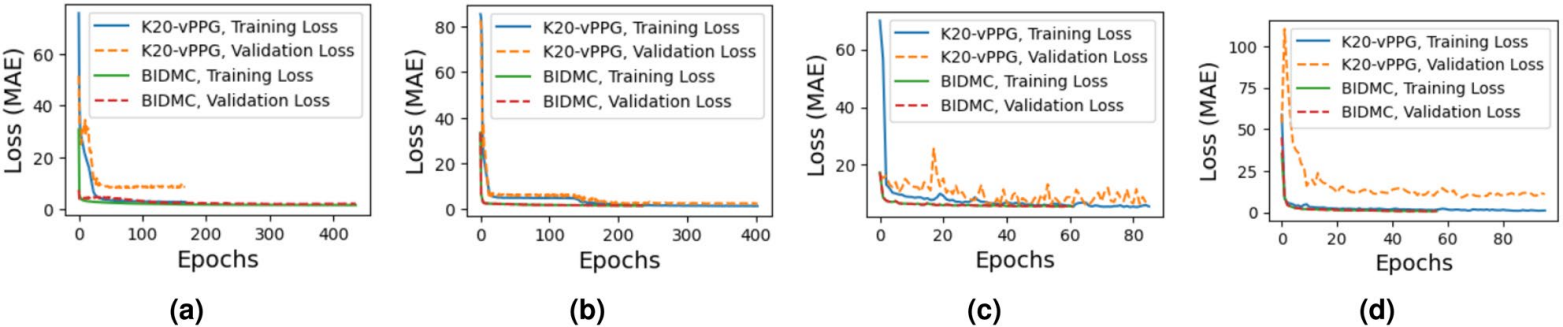
Training and validation loss of fine-tunned (**a**) CNN-Net, (**b**) MT-Net, (**c**) ViT-Net, (**d**) ResNet50 models using K20-vPPG and BIDMC datasets.

After finding the best window size, we train our DL models separately for each vital (but the curves that capture the decreasing trend of their training and validation losses against the number of epochs are omitted, due to space constraints). Next, we train the four models jointly using all vitals. Figure 6 shows their training and validation losses when trained using the two datasets, i.e., K20-vPPG and BIDMC. More specifically, Fig. 6 shows that the training and validation losses of CNN-Net and MT-Net saturate after 200 epochs. However, the training and validation losses saturate earlier (in about 20 epochs), for ViT-Net and ResNet50 models.

Table 3 does the detailed performance comparison of all of our DL models when they are tested in a subject-wise manner on LESSO data, for the following scenarios: (i) when the models are trained jointly, (ii) when the models are trained in a vital specific configuration. Overall, the performance of models trained specifically for one vital is superior to the models trained using all vitals simultaneously. Further, we note that MT-Net outperforms all the other competitor DL models. Table 3 also provides detailed results due to use of Vitals-CLIP in the querying mode to estimate the vitals. We see that Vitals-CLIP outperforms CNN-Net and ResNet50 in the estimation of HR when trained in joint training configuration. Also, Vitals-CLIP outperforms all models in SpO2 estimation for the BIDMC dataset. Also, here it is worth noting that almost all models fail to estimate SpO2 using a single PPG signal of the BIDMC dataset. Note that SpO2 estimation requires two PPG signals recorded using two lights of different wavelengths. The reason behind the failure of most of the models to predict SpO2 is that BIDMC dataset only has one PPG signal available in contrast to the video PPG where three PPG signals are available e.g. from RGB channels. Here it is worth noting that Vitals-CLIP outperforms other methods in SpO2 estimation due to the fact that Vitals-CLIP works in querying mode.

**Table 3:** MAE performance of our DL models for vitals estimation, on the LESSO data of two datasets (K20-vPPG and BIDMC).

Vital specific training configuration												
	K20-vPPG						BIDMC					
	HR		SpO2		RR		HR		SpO2		RR	
	$\mu$	$\sigma$	$\mu$	$\sigma$	$\mu$	$\sigma$	$\mu$	$\sigma$	$\mu$	$\sigma$	$\mu$	$\sigma$
DL models												
CNN-Net	2.22	4.04	0.64	0.61	3.95	2.78	4.10	2.91	5.91	5.21	2.85	2.24
MT-Net	2.07	4.00	1.99	1.53	3.58	2.45	4.19	2.48	6.12	7.33	2.67	2.67
ViT-Net	4.11	3.74	2.22	2.01	4.31	3.36	4.36	4.3	3.01	6.59	2.44	2.24
ResNet50	3.32	3.83	2.26	1.91	2.62	3.57	4.20	6.02	6.66	6.14	4.99	3.82
Joint training configuration												
CNN-Net	5.99	6.28	1.05	1.16	3.46	2.92	4.78	16.63	93.75	17.57	3.10	2.80
MT-Net	5.62	6.22	0.64	0.61	3.16	2.72	4.64	16.97	5.44	16.85	2.54	1.98
ViT-Net	5.88	5.00	2.35	2.01	3.20	2.7	5.88	5.00	2.35	2.01	3.20	2.70
ResNet50	6.17	7.92	1.61	0.54	2.62	3.57	4.20	16.56	6.38	6.76	4.99	3.82
CLIP configuration												
Vitals-CLIP	5.91	4.22	2.01	4.03	3.11	3.57	4.43	4.01	2.31	2.02	3.63	4.01

The DL models are tested subject-wise on the PPG data of each subject in LESSO data, and MAE is recorded for each subject. Then, $\mu$ and $\sigma$ represent the mean and standard deviation of MAEs, calculated across the subjects in LESSO data.

At this point, it is worth reminding the reader that we have used LESSO data to evaluate the performance of our DL models in Table 3 because this testing method is a superior indicator of the generalization capabilities of a DL model^53^. Nevertheless, we note that a large portion of the literature utilizes MAE of test dataset as performance metric. Therefore, we do a performance comparison of our work with the related work based upon MAE of test dataset in Table 6. We learn from Table 6 that the MAE performance of our DL models on test data is quite competitive. That is, for HR/SpO2/RR estimation, the MT-Net model/CNN-Net model/ViT-Net model achieves the best accuracy of 1.74 bpm/1.66%/0.89 brpm among all the DL models that we have implemented, while the state-of-the-art achieves 1.4 bpm/1.1%/0.67 brpm accuracy, respectively.

**Figure 7:**
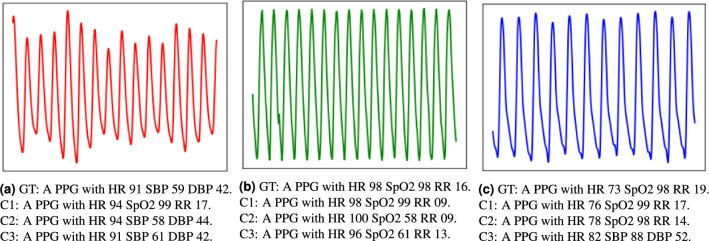
Ground truth caption and top three matching captions predicted by Vitals-CLIP for the PPG signals from (**a**) PulseDB dataset, (**b**) BIDMC dataset, (**c**) K20-vPPG dataset. Here GT stands for ground truth, while C1, C2, C3 are top three matching captions for which the dot product of text and PPG embedding are highest, second highest and third highest respectively. HR is measured in beats/min, RR is measured in breaths/min, SpO2 is measured in percentage, SBP and DBP are measured in mmHg.

Figure 7 shows three different query-PPG signals from three different datasets and their corresponding top three matching captions and the ground truth caption. After getting the matching caption the value of labels extracted from caption strings and then taken average. From the figure it is worth noting that the matching caption can sometimes also gives the values of systolic blood pressure (SBP) and diastolic blood pressure (DBP). This is due to the fact that the PulseDB dataset contains three two vitals HR and blood pressure (BP) and some time query PPG signal matches with a caption of the PulseDB dataset. Here it is worth mentioning that due to the unavailability of BP labels for the vPPG and BIDMC datasets, we could not add SBP and DBP vitals estimation performance in the table. However, it can be deducted from the nature of captions that the performance of SBP and DBP estimation would be similar to the other vitals.

#### Computational complexity of our DL models

Table 4 provides a concise analysis of memory and computational cost of each of our DL models. We observe from Table 4 that the memory requirement and the computational complexity of MT-Net and CNN-Net models are much lower than the rest of the models, making their implementation on resource-constrained devices such as Arduino feasible. On the other hand, the remaining DL models require relatively bigger memory and computational resources, therefore, they are more suitable for devices such as smartphones. One more important observation is as follows. Since we eventually prune (i.e., shred neurons and hidden layers of) our DL models in order to reduce their size, memory requirements and computational complexity, in order to port them onto a smartphone Android app, the computational complexity of the converted models reduces even further.

**Table 4:** Computational complexity and memory-requirements of the proposed DL models.

Models	Memory and computational cost		
	Memory	Parameters	FLOPs
CNN-Net	14.37 KB	3.673 K	25.434 K
MT-Net	35.04 KB	8.969 K	17.671 K
ViT-Net	82.62 MB	21.66 M	41.63 M
ResNet50	89.99 MB	23.58 M	47.16 M
Vitals-CLIP	28.48 MB	7.46 M	6.194 M
P2E-Net	326.95 KB	83.7 K	43.12 K

FLOPs stands for floating-point operations (i.e., additions and multiplications).

#### Lessons learned from android app development

After the customization of the DL models of Vitals-Net, we convert them into smartphone-compatible models using the TensorFlow lite package (note that Vitals-Net collectively refers to CNN-Net, MT-Net, ViT-Net, and ResNet50, in this work). We, then, make a customized app for vitals estimation using these Vitals-Net converted to TensorFlow lite model. To our surprise, we learned from this experience that it was not our DL models which was the performance bottleneck, rather it was the following:

- The first bottleneck is the respiratory rate (RR) process itself. RR, by definition, is a very slow process, i.e., the typical breathing rate of a healthy person is between 15 and 20 breaths/min. This means there is approximately one breath every 3 s or so. This phenomenon forces us to record a relatively longer video of the fingertip, say, of duration 30 s, so that we can capture at least 10 breathing cycles in our video-PPG data, in order to make the learning process of our DL models efficient. This constraint of recording a relatively longer video then leads to the second bottleneck below.
- The second bottleneck is the very first pre-processing step that converts the video-PPG data to the PPG signal through pixel-averaging of each frame. Let’s illustrate this by means of an example. Assuming a camera frame rate of 30 frames/s, with each frame having $1024 \times 1024 \approx 1$ million pixels, and a video-PPG data of duration 30 s, the smartphone needs to do pixel-averaging for $30 \times 30 = 900$ frames in total (i.e., 0.9 billion additions in total, in lieu of pixel-averaging).

**Figure 8:**
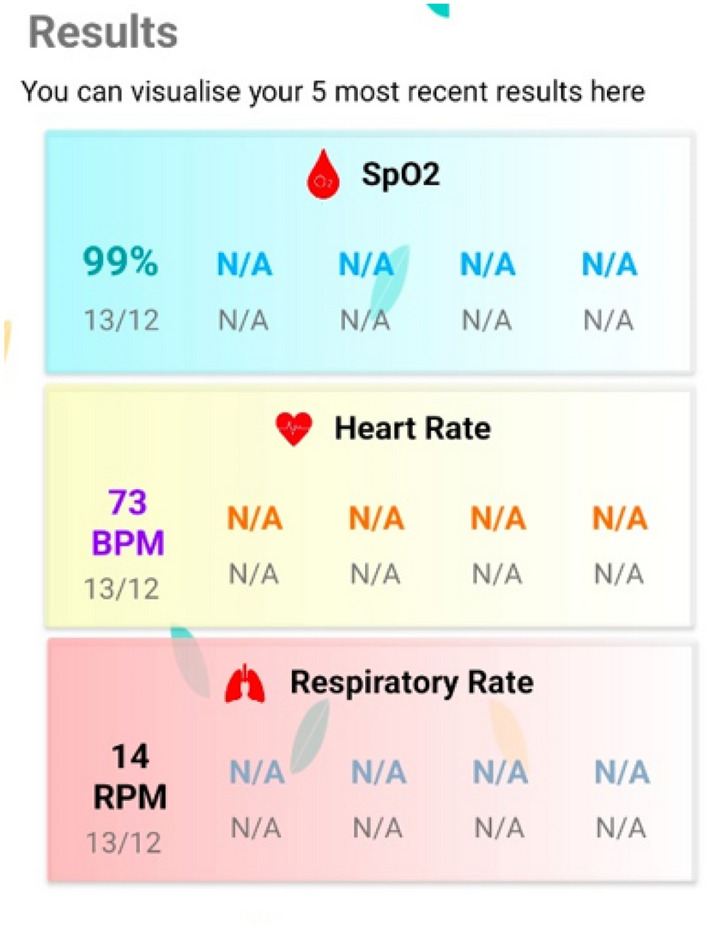
Screenshot of results panel of our custom Android app developed using the proposed Vitals-Net framework.

We alleviated the second bottleneck in our android app by processing a subset of each frame, the so-called region-of-interest (ROI), basically a rectangular patch of pixels in the center of each frame. This sheds roughly half of the computational complexity of video-PPG to PPG translation process. After extensive testing, trouble-shooting, fine-tuning, we conclude our android app development campaign with the affirmitive claim that the standard smartphones are indeed capable of sustaining the computational requirements of our proposed method. For example, on a Vivo smartphone, model V2024, having android 10, 2 GHz Snapdragon 665 octa-core processor, 4 GB of RAM, and 128 GB of storage, our custom android app predicts the HR and SpO2 of a subject in about 1 minute, while it predicts the RR in about 3 min. Figure 8 shows a screenshot of the results panel of our custom-designed Android app that lets a user record a video of his/her fingertip, preprocesses the raw video data, feeds it to our proposed DL models, and eventually displays the results.

## Single-lead ECG synthesis from video-PPG

This section aims to reconstruct/synthesize a single-lead ECG signal from a PPG signal which itself has been extracted from the video-PPG data that is acquired by placing the fingertip on the rear camera of a smartphone. More precisely, we aim to reconstruct the ECG Lead-I, as per nomenclature for a standard 12-lead ECG system. Mathematically, the problem at hand is to find a mapping from a function *x*(*t*) to another function *y*(*t*), and vice versa. This (translation or regression) problem is indeed feasible due to the fact that the two signals (PPG and ECG) are highly correlated as they both capture the same cardiac activity at a sub-cardiac cycle resolution. Further, the morphology of the two signals is tightly binded to each other, from one cardiac cycle to another (e.g., the R-peak of the ECG corresponds to the diastolic peak of the PPG signal and more). Next, as we did in the previous section, we first describe the crucial pre-processing steps, followed by the details of our proposed deep learning-based model (P2E-Net), followed by the performance evaluation of the proposed method.

### Pre-processing stage

Pre-processing of both the reference single-lead ECG signal and the PPG signal (extracted from the video-PPG data) plays an important role in the efficient training of the P2E-Net. That is, it not only eases the training process but also improves the quality of the reconstructed single-lead ECG waveform. Thus, we segment the raw ECG and PPG signals, and pass them through a wavelet-filtering block that removes baseline drift, high-frequency noise, and artifacts induced by ambient light from both ECG and PPG signals (see Fig. 2) (We note that though the basic filters like moving average (MA) filter excel at eliminating high-frequency noise, but they fall short in removing prominent (low-frequency) baseline drifts and artifacts from the raw PPG and ECG signals. In contrast, the wavelet-based approach efficiently eliminates baseline, artifacts and noise from the raw PPG and ECG signals, which improves the performance of the (PPG and ECG) peak detection algorithm during the training and testing phase of our P2E-Net model. Thus, we feed Wavelet-denoised signals to our P2E-Net model, which results in enhanced performance for 1-lead ECG synthesis.).

**Figure 9:**
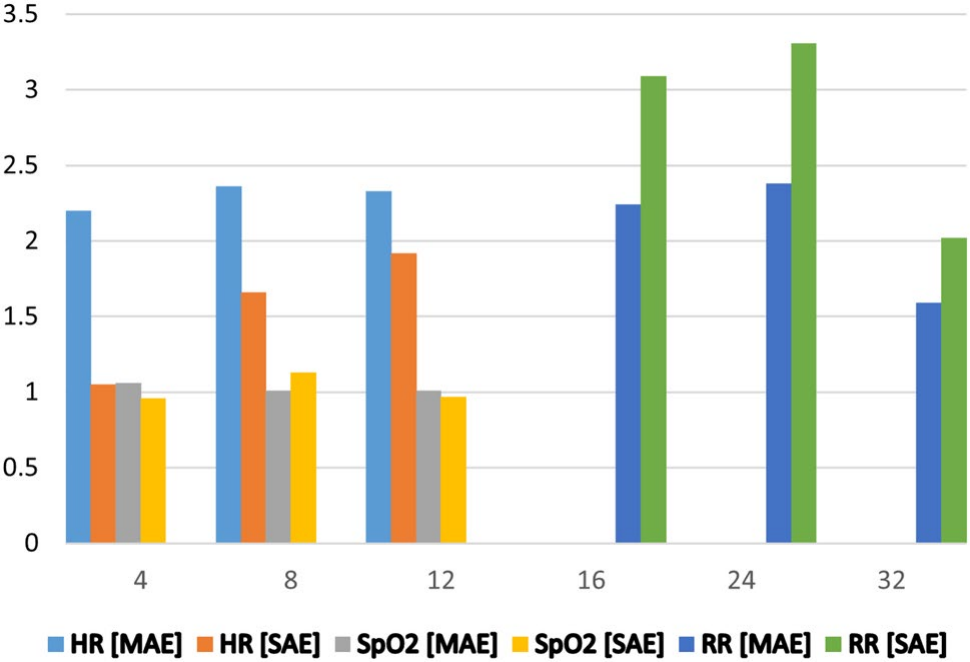
Impact of window size on vitals estimation (x-axis represents window size, the y-axis represents MAE and SAE).

Next, some additional preprocessing steps for P2E-Net model, that were not required by the Vitals-Net models, are discussed below (Fig. 9).

#### Peaks and valleys detection

During this step, the peaks and valleys of the ECG and PPG signal are detected which later help in signal de-trending and synchronization. Specifically, during the training phase of P2E-Net model, both the R-peaks of ECG and diastolic peaks of PPG are detected, using the TERMA algorithm^56^ (see Fig. 16). On the other hand, during the testing phase of P2E-Net model, only the diastolic peaks of PPG need to be detected. In other words, during the testing phase of P2E-Net model, detection of R-peaks of ECG and synchronization between PPG and ECG is not required.

#### Signal synchronization and de-trending

For detrending the PPG and ECG signals, we first construct the upper and lower envelopes of the two signals by interpolating between the peaks and valleys points respectively (see Fig. 3b). More specifically, we use the ‘spline(.)’ function of Matlab to construct upper and lower envelopes, using detected peaks and valleys points of the two signals. Then, to detrend the two signals, we first subtract the lower envelope from the PPG/ECG signal that de-trends the PPG/ECG signal from the lower side. Then, we divide the PPG/ECG signal by a new upper envelope that we obtain by subtracting the lower envelope from the upper envelope. We then use this new upper envelope to de-trend the PPG/ECG signal from the upper side. This way, we get a de-tended signal having magnitude between 0 and 1. We further give the detrended signal an offset of 0.5, in order to center it at zero (see Fig. 3c). Once the PPG/ECG signals are de-trended, they are synchronized/aligned by using diastolic peaks of PPG and R-peaks of ECG signal as reference points.

### The proposed P2E-Net framework

**Figure 10:**
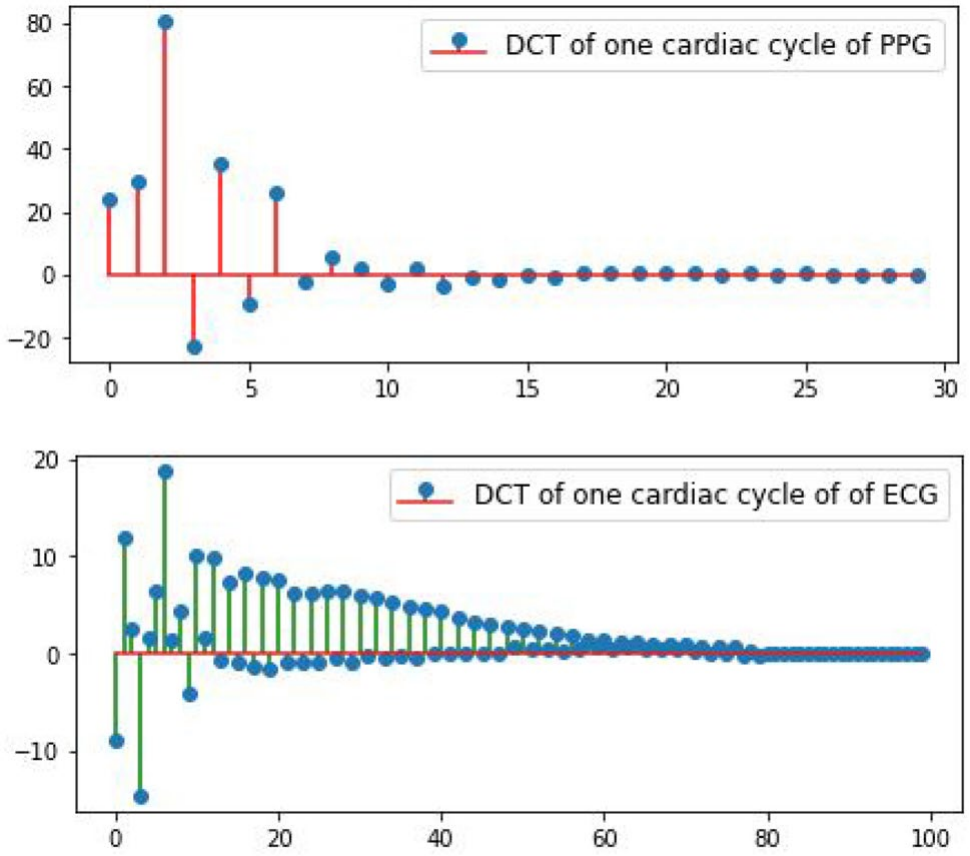
DCT coefficients of the PPG signal and the single-lead ECG signal (one cardiac cycle only). The proposed P2E-Net framework learns the regression between the two sets of DCT coefficients.

The proposed approach aims to map one cardiac cycle of vPPG to one cardiac cycle of ECG, as illustrated in Fig. 10. To achieve this, two configurations of P2E-Net are trained and tested, namely, fully connected neural networks and ridge regression models-based configurations. These configurations differ based on the type of input/output layers or the network architecture. In the Ridge regression configuration, P2E-Net consists of an input layer, a discrete cosine transform (DCT) layer, a regression layer, an inverse DCT (IDCT) layer, and an output layer. On the other hand, in the feedforward neural network (FFNN) configuration, P2E-Net includes the same input, DCT, IDCT, and output layer, but two hidden layers replace the linear regression layer of the Ridge regression configuration. The hidden layers consist of a fully connected layer with ’selu’ activation function followed by a batch normalization layer. Both hidden layers and FFNN configuration also apply $L_1$ regularization to avoid overfitting.

**Figure 11:**
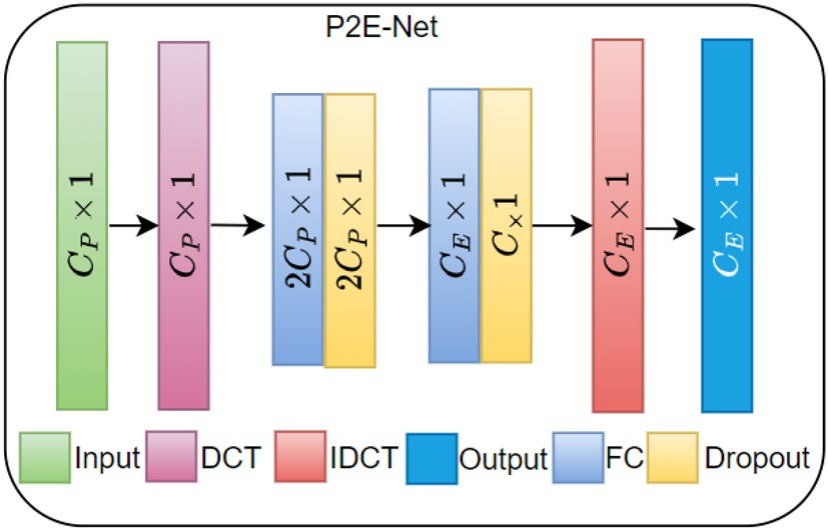
P2E-Net model architecture in which each fully connected (FC) layer uses ’tanh’ activation and $C_P = 150$ and $C_E = 150$.

The input layer of P2E-Net is, actually, the output of pre-processing layer, as described earlier. In the training phase, the pre-processing layer output cycle-wise time-domain vPPG as well as reference/ground truth ECG denoted by $C_P \in {\mathscr {R}}^{L \times 1}$ and $C_E\in {\mathscr {R}}^{L \times 1}$ respectively. Then, the cycle-wise time-domain vPPG is fed to the DCT layer of P2E-Net. The DCT layer, then, computes the DCT coefficient $c_P \in {\mathscr {R}}^{L_P \times 1}$ that corresponds to the cardiac cycle of vPPG. At this stage, the DCT coefficients of the reference ECG cycle $c_E \in {\mathscr {R}}^{L_E \times 1}$ are also computed offline. The DCT coefficients $c_P$ are fed to the regression/hidden layers, based on the configuration of P2E-Net, that maps them to the DCT coefficients of reconstructed ECG denoted by $\hat{c_E} \in {\mathscr {R}}^{L_E \times 1}$. The DCT coefficients $c_E$ and $\hat{c_E}$ are used in the loss computation and optimization of the model. Figure 11 provides the complete architecture of the P2E-Net model.

During the training phase, the iDCT layer remains inactive but once the network is trained and ready to be tested, it activates and serves to construct the time domain ECG signal from predicted $\hat{c_E}$.The regression model similar to Ref.^45^, when trained, learns a linear mapping from the $c_P$ to the $c_E$. In contrast to regression, P2E-Net in FFNN configuration learns a non-linear mapping from vPPG DCT to the DCT of ECG. In the IDCT layer, the ECG signal is reconstructed.

#### Training of P2E-Net

The P2E-Net is trained for a maximum of 1000 epochs. All the trainable network parameters are initialized with Xavier initializer. The MAE loss function is used to optimize P2E-Net and for the optimization, Adam optimizer is used. In the FFNN configuration, a learning rate scheduler, named staircase exponential learning rate decay, with a decay rate of $\exp (0.1)$ is applied to accelerate the convergence and achieve better performance. Moreover, with the aim of avoiding the over-fitting of the model, we apply $L_1$ regularization of hidden layers. An early stopping with verbose 5 is also applied. The best model with the least validation loss is saved after every 5 epochs during training, to see the performance of the best-trained model on test data. Other common training hyperparameters used in these models are shown in Table 5.

**Table 5:** Values of other hyper-parameters for P2E-Vital-Net.

Hyper-parameters	P2E-Net	Vitals-Net
Dataset size	*d*	*N*
Validation data set size	*d*	$0.15*N$
Test data set size	0.1*d	$0.10*N$
LESSO data set	0.1*d	5 subjects
Training batch size	100	128
Maximum number of epochs	1000	1000
Initial learning rate	$10^{-3}$	$10^{-3}$
learning rate scheduler	Exponential decay	–
Scheduler type	Staircase	–
Learning rate decaying factor	exp(0.1)	0.1
Optimizer	Adam	Adam

Here $N = 28,938$ for pulseDB, $N = 8890$ for K20-vPPG, $N=22,550$ for BIDMC, and $d = 6380$.

### Performance evaluation

In order to evaluate the performance of P2E-Net, we performed experiments using two different datasets, i.e., we first used the BIDMC dataset and then the vIDEO-P2E dataset. In order to investigate the generalization capability of P2E-Net, we randomly selected five subjects that were excluded from the training and validation dataset. From the remaining subjects, we split each session into 80% and 20% for training and validation respectively. Then we evaluated the performance of the proposed method with left-over data. For the rigorous evaluation of P2E-Net, we used three performance metrics, namely Pearson correlation and Dirichlet distance, and MAE. The Pearson correlation coefficient and Dirichlet distance are defined as:

2 $$\begin{aligned} P_{corr}= & {} \frac{ ({{\textbf {x}}} - \mu _x)^T({{\textbf {y}}} - \mu _y)}{||{{\textbf {x}}} - \mu _x||_2 ||{{\textbf {y}}} - \mu _y||_2}, \end{aligned}$$

3 $$\begin{aligned} l_{dir}= & {} \min (\max _{i \in {\mathscr {Q}}}(d(x_i, y_i))), {\mathscr {Q}} = [1,N]. \end{aligned}$$

In Eq. (2), ${{\textbf {x}}}$ represents the reference ECG signal and $\mu _x$ represents its mean. Similarly, ${{\textbf {y}}}$ and $\mu _y$ represent the reconstructed ECG signal and its mean, respectively. In Eq. (3), the notation d(*) represents the Euclidean distance between two points. $x_i$ and $y_i$ are the $i^{th}$ elements of **x** and **y** respectively and ${\mathscr {Q}}$ is set of integers [1, *N*] where *N* is the length of **x**.

**Figure 12:**
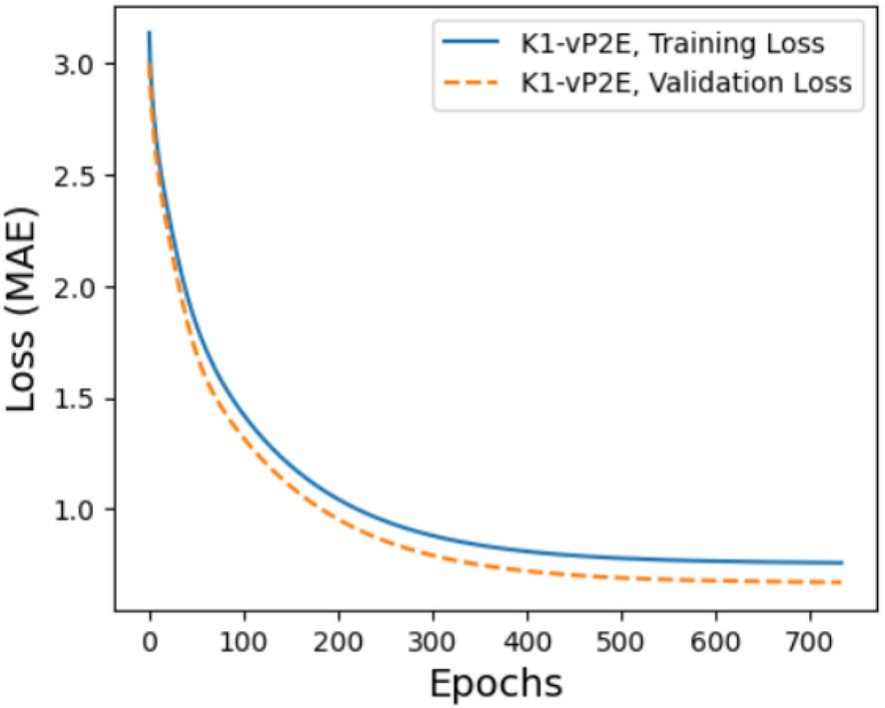
Training and validation loss of P2E-Net.

The P2E-Net model was trained for a maximum of 1000 epochs, but due to early stopping, the training stopped after just over 400 epochs when the MAE loss plateaued at 0.4 for training and 0.46 for validation, as shown in Fig. 12.

We then examined the impact of the number of vPPG DCT coefficients, and Fig. 15 shows that using a larger number of DCT coefficients improves ECG reconstruction performance in terms of all performance matrices, but not significantly. Overall, the proposed P2E-Net models can generate ECG signals with a mean absolute error below 0.1, a correlation with the reference ECG above 0.8, and a Dirichlet distance around 0.2 on average. It is worth noting that all these results are obtained using left-over datasets, which proves the efficacy of P2E-Net in terms of generalization capability (Table 6).

**Table 6:** MAE performance comparison of our work on vitals estimation with the most relevant related works on the test data (all works utilize video-PPG data from the fingertip).

Ref.	Vitals measured	HR (MAE)	SpO2 (MAE)	RR (MAE)
^13^	PR, SpO2, BP	1.4 bpm	1.1%	–
^14^	SpO2	–	2.02%	–
^15^	SpO2	–	3.5%	–
^18^	HR, SpO2	6.59 bpm	1.24%	–
^43^	RR	–	–	0.67 brpm
^44^	RR	–	–	0.89 brpm
Our models	Vitals Measured	HR (MAE)	SpO2 (MAE)	RR (MAE)
MT-Net	HR, SpO2, RR	1.74 bpm	1.73 %	1.41 brpm
CNN-Net	HR, SpO2, RR	1.89 bpm	1.66 %	1.79 brpm
ViT-Net	HR, SpO2, RR	3.41 bpm	2.64 %	0.89 brpm
ResNet50	HR, SpO2, RR	2.01 bpm	2.01 %	3.14 brpm
Viltals-CLIP	HR, SpO2, RR	4.31 bpm	1.96 %	3.01 brpm

bpm, and brpm stand for beats per minute and breaths per minute, respectively.

**Figure 13:**
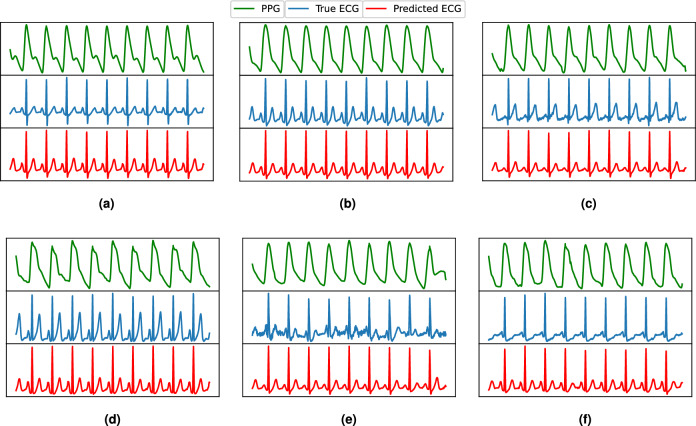
Some ECG reconstruction examples using the ridge regression method.

**Figure 14:**
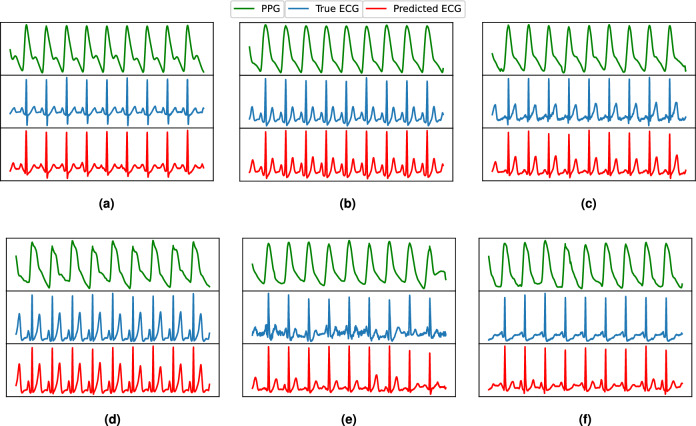
Some ECG reconstruction examples using the proposed P2E-Net model.

**Figure 15:**
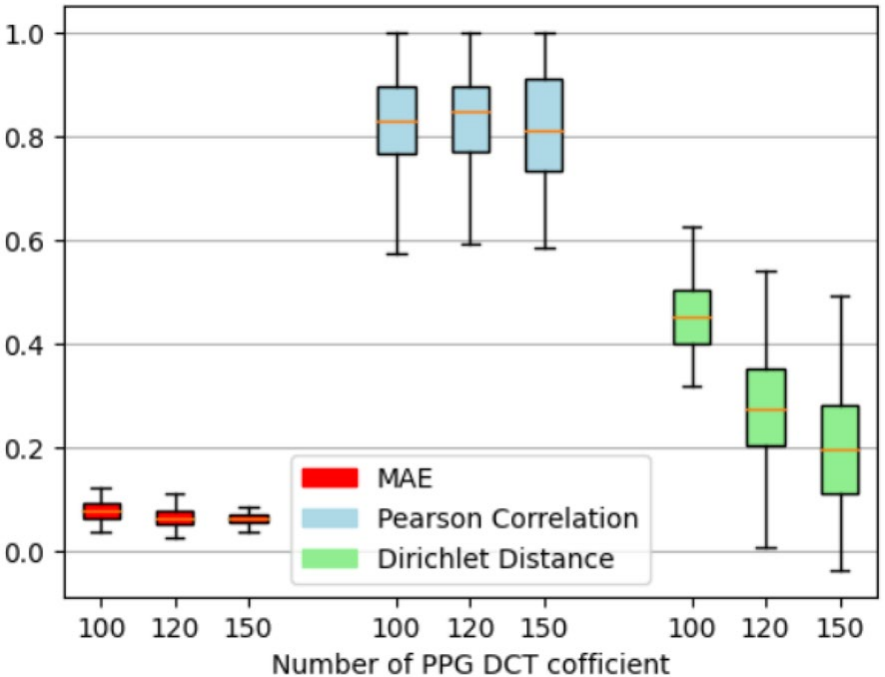
Impact of the number of DCT coefficients on the performance of proposed P2E-Net.

**Figure 16:**
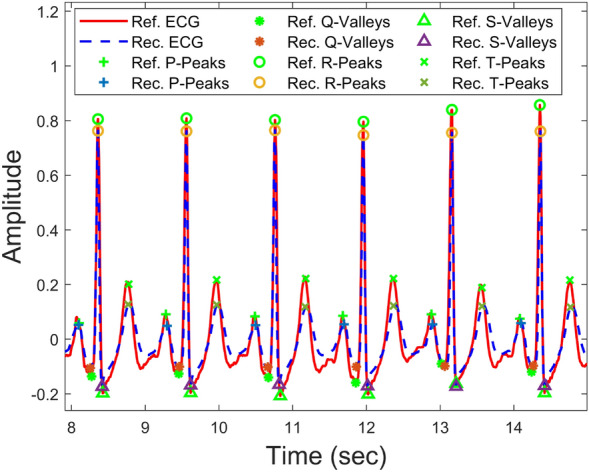
Peaks and valleys of reference and reconstructed ECGs.

As ECG signals are mainly characterized by the P, QRS, and T peaks, we performed a cardiac cycle-level investigation. We selected the ECG signals of five subjects from the left-over data and detected all peaks as shown qualitatively in Fig 16. The peak-level quantitative performance of P2E-Net is presented in Table 7, which shows that neural network-based models outperform the ridge regression model, and both methods efficiently reconstruct the ECG. Finally, Figs. 13 and 14 show the qualitative performance of the ridge regression method and FFNN-based method whereby the reconstructed ECG waveforms show a high morphological similarity with the reference ECG waveform (for a few chosen subjects) (Figs. 15, 16).

**Table 7:** Performance of P2E-Net. Mean and std. dev. of Peaks and Valleys of reconstructed ECG waveforms for five subjects.

Method	P-Peaks		Q-Valleys		R-Peaks		S-Valleys		T-Peaks	
	MMAE	MSAE	MMAE	MSAE	MMAE	MSAE	MMAE	MSAE	MMAE	MSAE
DCT+Ridge regression	0.0487	0.01996	**0.05722**	0.0243	0.0883	0.0419	**0.0850**	**0.0291**	0.1125	0.0319
DCT+FFNN	**0.0418**	**0.0177**	0.0612	**0.0177**	**0.059**	**0.0381**	0.09516	**0.0303**	**0.1044**	**0.0275**

Significant values are in bold.

In summary, the P2E-Net efficiently reconstructs ECG signals with a shallow FFNN model, making it highly suitable for deployment on smartphones.

### Some important remarks regarding proposed P2E-Net model

Before we conclude the discussion on our P2E-Net model, it is worth emphasizing the following aspects regarding preprocessing of the P2E-Net model:

- The training phase of P2E-Net model, the test phase of P2E-Net model, (and the training phase of Vitals-Net models) each have distinct requirements for the preprocessing steps (see Fig. 2). More precisely speaking, the training of P2E-Net model requires a preprocessed dataset that requires the following two additional steps (when compared to the preprocessing steps needed to prepare the training data for the Vitals-Net): (i) R-peaks detection of ECG, (ii) Peaks synchronization of ECG and PPG signals. These two steps require some manual effort, especially for those ECG signals in which R-peaks are not prominent, to make the preprocessed dataset as efficient as possible. However, recall that the testing phase of P2E-Net aims to translate an input PPG signal into a synthetic ECG signal. Therefore, we reach to the pleasing conclusion that the test phase of our P2E-Net model neither requires ECG peaks detection, nor the PPG and ECG signal synchronization. In other words, the bulk of the preprocessing is done offline during the training phase when the two datasets (i.e., K20-vPPG and K1-vP2E) are meticulously prepared to train the Vitals-Net and P2E-Net models. Therefore, it is safe to assert that the performance of our P2E-Net model does not depend on the aforementioned additional preprocessing steps during the testing in the real-time.
- One also needs to consider the impact of potential changes in the morphology of the ECG signals (due to different cardiac diseases and different body sites for ECG measurement electrodes), on the performance of R-peaks detection algorithm. However, it is again imperative/helpful if we look at the test phase and training phase of our P2E-Net model separately. Recall that the testing phase of our P2E-Net model needs to do the peak detection of the input PPG signal only (and not the ECG signal, as ECG needs to be rather synthesized by our model). Then, it is a known fact that though the morphology of a PPG signal changes due to a number of factors, its peaks still remain prominent, resulting in high PPG peak detection performance. The training phase of P2E-Net indeed requires efficient peak detection of ECG. Thus, for some morphologies of ECGs where R-peaks are not prominent, or when ECG signals have notable artifacts or baseline that cannot be removed completely, the classical peak detection approach may fail. In such cases, one could manually adjust the threshold to efficiently detect the R-peaks where possible. We believe this is a satisfactory workaround since the training is performed offline.

## Discussion

Previous two sections described our DL-based approach for vitals estimation and single-lead ECG synthesis using a smartphone alone, as well as the selected results in great detail. This section discusses both the promise of our work as well as its limitations (i.e., directions for future work). We first summarize the promise of this work as follows:

- *Pioneer work on single-lead ECG synthesis using a smartphone* To the best of our knowledge, this is the first work that reconstructs a single-lead ECG from a video-PPG signal acquired from a smartphone, without any alterations, i.e., without any additional sensors or electrodes externally attached to the smartphone.
- *A step towards patient-centric healthcare systems* This work enables rapid self-testing of various important body vitals (i.e., heart rate, respiratory rate, blood oxygen saturation level), using one’s own smartphone. This opens the door for the realm of patient-centric healthcare whereby the chronic patients, palliative care patients, people in remote areas (mountains, deserts), people in marine/sea expeditions, people in harsh climates (e.g., arctic), people in religious congregations (e.g., Hajj, Umrah, Khumb, etc.) could monitor and keep track of their body vitals. The proposed method could also revolutionize the symptom monitoring and screening methods for covid19 suspects and patients (as it allows self-screening for covid19 symptoms at public places, e.g., shopping malls, airports, concerts, etc.).
- *Early diagnosis of cardiac diseases* This work enables an off-the-shelf smartphone to provide a single-lead ECG. Thus, it enables rapid self-testing for atrial fibrillation detection using one’s own smartphone. Once atrial fibrillation is detected by the smartphone, it will prompt the user to visit a hospital (or send an SOS message to the nearby rescue health facility). Since atrial fibrillation is a risk factor for stroke and heart failure, a timely detection of atrial fibrillation could in turn lead to early diagnosis of a range of cardiovascular diseases at a tertiary healthcare facility. Therefore, the proposed method has the potential to reduce the burden at tertiary healthcare facilities. Additionally, early diagnosis of a variety of cardiovascular diseases will lead to early intervention by the cardiac physicians which will in turn lead to reduction in health care (and health insurance) costs, and will potentially save many precious lives.
- *A step towards generative AI for medical data* We do hope that our two custom datasets (K20-vPPG and K1-vP2E) will provide aid to the emerging generative AI methods that aim at generating synthetic (but sophisticated) medical data. Further, the two datasets will serve as benchmark datasets to test the generalization capability, accuracy and robustness of future deep learning methods for vitals estimation and single-lead ECG synthesis. Similarly, we hope that our DL models will serve as baseline models, in order to design future AI-based solutions that aim to monitor an increased number of human physiological parameters using a smartphone.
- *Smartphone as a DIY health diagnostic tool inline with the UN SDGs* This work is one-step forward to turn a smartphone into a do-it-yourself (DIY) health diagnostic tool. Thus, this work is anticipated to help achieve the sustainable development goals (SDG) 3 & 10 set by the United Nations (UN) that aim at health and well-being and reducing the inequality within and among countries, respectively.

Having described the strengths of our work, we now turn to its limitations (i.e., the directions for the future work):

- *Data distribution-dependent performance of DL models* Like all other DL models reported in literature, our DL models are also dependent upon the distribution of the dataset. Thus, our DL models could undergo a performance degradation when exposed to a new dataset (that is similar but not the same as the original dataset our DL models are trained on). For example, the SpO2 labels in our dataset (K20-vPPG) fall in a narrow range of (90–100)%, due to the fact that this dataset was collected from the healthy young subjects only. Therefore, our proposed DL models do not promise to generate accurate SpO2 predictions for the patients with severe respiratory disease (e.g., pneumonia, covid19, etc.) whose SpO2 could be as low as 70%. The solution to this problem is twofold. (1) More data needs to be collected, but this time from real patients with cardiac and respiratory diseases. (2) With the new dataset constructed, one could do transfer learning (by keeping the weights and biases of neurons in all the layers in our neural network models frozen, except last 1–2 layers) in order to learn the new distribution of the data.
- *Difficulty in acquiring medical data* Continuing with above argument, another important challenge is the difficulty in acquiring medical data (e.g., due to considerable waiting time for approvals from ethical boards, due to lack of willingness of patients to volunteer for data collection, due to more emphasis by clinicians and hospitals on clinical aspect and less emphasis on research aspect, etc.).
- *High time for generative AI for medical data* Continuing with above argument, practical difficulties in acquiring medical data call for construction of synthetic (but realistic) datasets. This dream has become feasible very recently due to the rise of generative AI methods. Indeed, efforts have begun already to use generative AI tools (e.g., auto-encoders, generative adversarial networks) to generate synthetic but reliable medical data.
- *The pursuit for clinical trials:* Our DL models and the resulting Android app need to undergo extensive clinical trials in one or more hospitals in order for us to validate the performance of our DL models on real patients. This is indeed one of the long-term objectives of this work.
- *Edge computing or cloud computing?* Not all the DL models proposed in this work (and previous works) could be implemented on the resource-constrained devices. For example, ResNet50 model and Vitals-CLIP model proposed in this work have higher memory requirements and higher computational complexity compared to the other models (see Table 4). This makes their deployment on resource-constrained devices (e.g., Arduino, raspberry pi, low-end smartphones etc.) difficult. However, if latency is not an issue, then cloud computing could be one viable solution for the resource-constrained devices. Similarly, high-quality synthesis of a single-lead ECG in this work requires efficient preprocessing (which involves wavelet filtering, peaks detection and signal detrending), which increases the computational complexity of our approach, yet the situation remains under control when we port our method to an Android app. For the sake of records, we have been able to successfully implement the lighter version of our CNN-Net model onto a regular smartphone by means of Tensorflow lite framework. That is, in a real-time setting, our custom android app computes the HR and SpO2 of a subject about a minute, while it takes about three minutes to compute the RR of the subject. This demonstrates that some of our proposed DL models are quite suitable for edge AI computing on resource-constrained devices.

Finally, following points are also worth mentioning. (1) The proposed solution is probably best claimed as a self-help or self-testing tool, i.e., it is not a replacement for the gold-standard medical devices (e.g., traditional contact-based 12-lead ECG machines) in the hospital. (2) Once the clinical trials of our proposed solution are over and successful, the next immediate and logical step is to seek approval from the food and drug authority (FDA). Such approval will facilitate our proposed solution to reach its true potential (to help millions of people around the globe).

## Conclusion

This work demonstrated the feasibility of using a smartphone as an initial diagnostic tool to measure one’s body vitals, i.e., pulse rate, SpO2, and respiratory rate, and a single-lead ECG. A number of custom-built CNNs and FFNNs (including a vision transformer and a CLIP model) were implemented to extract the body vitals as well as the single-lead ECG from the video-PPG signal recorded from the rear camera of the smartphone. Rapid self-testing of body vitals allows ubiquitous monitoring of one’s well-being (e.g., self-monitoring for covid19 symptoms). Similarly, rapid self-acquisition of a single-lead ECG allows early detection of atrial fibrillation (abnormal heartbeat), which in turn could enable early intervention in response to a range of cardiovascular diseases, and could help save many precious lives. Overall, our work has the potential to revolutionize the healthcare systems as it could reduce the burden on healthcare facilities and could lead to a reduction in health insurance costs. This work invites smartphone manufacturers and Android app developers to deliberate and standardize algorithms to measure body vitals and single-lead ECG, as well as governments to devise policies and guidelines for the following use case scenarios: remote healthcare (i.e., people living in remote and far-away areas), patient-centric healthcare (i.e., chronic and palliative care at-home patients), mobile health (i.e., monitoring of well-being of various long-journey expeditions, e.g., sea/marine expeditions), fitness, and sports, etc.

## Data Availability

The two custom datasets (K20-vPPG and K1-vP2E) used and/or analysed during this research study are available from the corresponding author on reasonable request.
